# *Plants* 2021 Best Paper Award

**DOI:** 10.3390/plants10061173

**Published:** 2021-06-09

**Authors:** Plants Editorial Office

**Affiliations:** MDPI, St. Alban-Anlage 66, 4052 Basel, Switzerland; plants@mdpi.com

*Plants* is instituting the Best Paper Awards to recognize the outstanding papers published in the journal. As the editorial office of *Plants*, we are pleased to announce the winners of the *2021 Plants* Year Best Paper Awards.

Papers published in 2019 were preselected by the *Plants* Editorial Office based on the number of citations and downloads from the website. The winner nominations were made by a selection committee, which was chaired by the Editor-in-Chief, Prof. Dr. Dilantha Fernando and supported by seventeen Editorial Board Members. The four top-voted papers, in no particular order, have won the 2021 *Plants* Best Paper Award:

## 1. Review Paper Award

**Plant Disease Detection and Classification by Deep Learning** [1]

Muhammad Hammad Saleem, Johan Potgieter and Khalid Mahmood Arif

*Plants***2019**, *8*(11), 468; doi:10.3390/plants8110468

Available online: https://doi.org/10.3390/plants8110468

**Figure 1:**
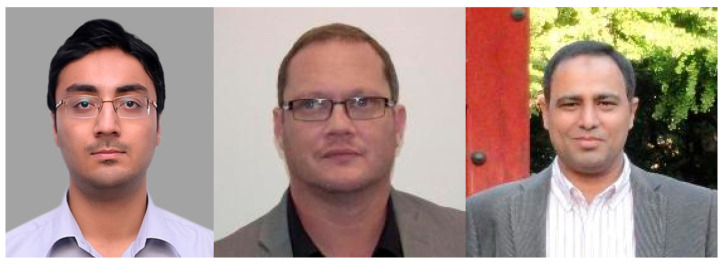
Muhammad Hammad Saleem, Johan Potgieter and Khalid Mahmood Arif (from left to right).

Precise identification of plant disease is important for the healthy growth of plants to meet the ever-growing food demand. In this regard, our group (Figure 1) is actively involved in deep learning (DL)-based plant disease detection and classification techniques. This review article presents the recent developments of DL models for plant disease detection and classification. The overall steps to implement the DL-based plant disease identification are explained. Many visualization techniques/mappings are presented to recognize the symptoms of plant disease. A comprehensive summary containing the performance of various state-of-the-art DL architectures tested on different datasets is provided. Similarly, the impact of advanced imaging techniques (such as hyperspectral imaging) with DL models are discussed. Moreover, this review highlights the research gaps in order to get a clearer/more transparent detection of disease in the plant species.

## 2. Research Article Awards

**Involvement of Phenolic Acids in Short-Term Adaptation to Salinity Stress is Species-Specific among Brassicaceae** [2]

**Figure 2:**
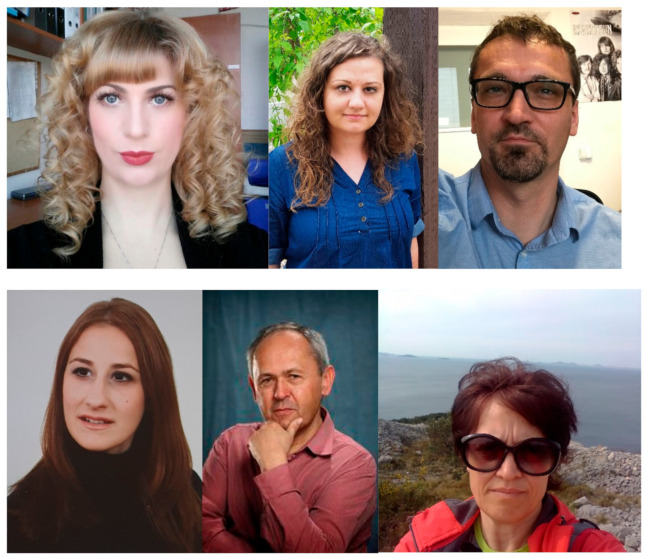
Ida Linić, Dunja Šamec, Jiří Grúz, Valerija Vujčić Bok, Miroslav Strnad and Branka Salopek-Sondi (from up to down and from left to right).

Ida Linić, Dunja Šamec, Jiří Grúz, Valerija Vujčić Bok, Miroslav Strnad and Branka Salopek-Sondi (Figure 2)

*Plants* **2019**, *8*(6), 155; doi:10.3390/plants8060155

Available online: https://doi.org/10.3390/plants8060155

Climate changes and associated abiotic stresses are a serious problem for agriculture. Elevated soil salinity is one of the most damaging abiotic stressors to plants, causing disruptions in physiological, biochemical and metabolic processes important for plant growth and consequently leading to a significant reduction in crop yield. Therefore, the study of the mechanisms of salinity tolerance in plants is of great importance. Our study highlights the role of phenolic acids in salinity tolerance in the plant family Brassicaceae. The highest levels of phenolic acids, especially hydroxycinnamic acids, were determined in the more tolerant kale and white cabbage compared to the more sensitive Chinese cabbage. We found that phenolic acids are species-specific in Brassicaceae and that some of them may be involved in stress tolerance.

**Isoform-Specific NO Synthesis by Arabidopsis thaliana Nitrate Reductase** [3]

**Figure 3:**
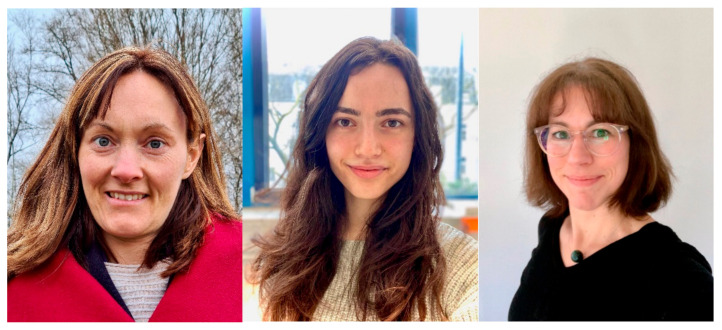
Marie Agatha Mohn, Besarta Thaqi and Katrin Fischer-Schrader (from left to right).

Marie Agatha Mohn, Besarta Thaqi and Katrin Fischer-Schrader (Figure 3)

*Plants* **2019**, *8*(3), 67; doi:10.3390/plants8030067

Available online: https://doi.org/10.3390/plants8030067

Nitrate reductase is the key enzyme in the nitrate assimilation pathway of plants, and is also suggested to be a major enzymatic source of the signaling molecule nitric oxide, produced by nitrite reduction. Arabidopsis thaliana possesses, like many plants, two isoforms of nitrate reductase, NIA1 and NIA2. In our study, we systematically compared both NIA isoforms, with a focus on their kinetic properties, and our data suggest that both NIA1 and NIA2 fulfill distinct functions in plant physiology. Based on their enzymatic characteristics, NIA1 is likely to be involved in cellular signaling, due to its significant reduction of nitrite to nitric oxide, whereas NIA2 is considered to function primarily in nitrogen metabolism, reducing nitrate to nitrite.

**GWAS for Starch-Related Parameters in Japonica Rice (*Oryza sativa* L.)** [4]

**Figure 4:**
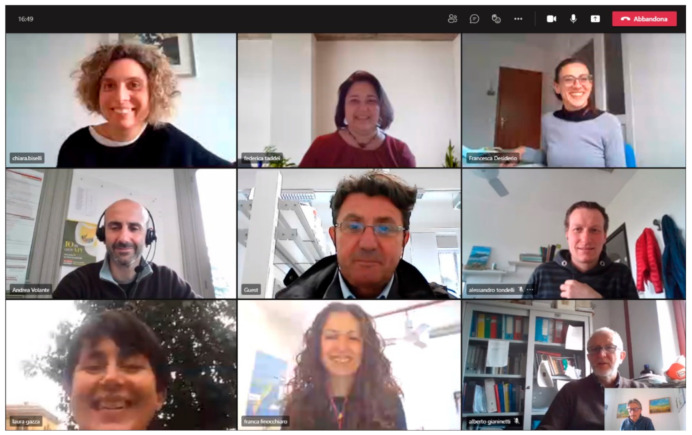
Chiara Biselli, Federica Taddei, Francesca Desiderio, Andrea Volante, Giampiero Valè, Alessandro Tondelli, Laura Gazza, Franca Finocchiaro, Alberto Gianinetti and Luigi Cattivelli (small picture) (from up to down and from left to right).

Chiara Biselli, Andrea Volante, Francesca Desiderio, Alessandro Tondelli, Alberto Gianinetti, Franca Finocchiaro, Federica Taddei, Laura Gazza, Daniela Sgrulletta, Luigi Cattivelli and Giampiero Valè (Figure 4)

*Plants* **2019**, *8*(8), 292; doi:10.3390/plants8080292

Available online: https://doi.org/10.3390/plants8080292

Rice is one of the main staple foods for worldwide population. Economic development and lifestyle improvements have brought more concerns about rice quality, which is related to grain composition (starch composition in particular). For example, rice cooking properties are mainly affected by the percentage of amylose on total starch, referred to as apparent amylose content (AAC): the grains of high AAC varieties remain firm and separate after cooking, while low AAC genotypes end up tender, glossy and cohesive. Another important component of rice grain is resistant starch (RS). RS is not digestible in the stomach and small intestine and remains intact in the colon, where it acts as a prebiotic. The beneficial health effects of RS consist of the improvement of insulin sensitivity, the decreasing of postprandial blood glucose and the prevention of colon rectal cancer. It also may assist in the prevention of metabolic syndrome, and could be a satiety agent.

In the paper “GWAS for Starch-Related Parameters in Japonica Rice (*Oryza sativa* L.)”, a genome wide association scan (GWAS) was performed on a collection of 115 japonica rice accessions, genotyped via genotyping by sequencing (GBS), with the aim to expand the knowledge of the loci implicated in the determination of AAC and RS. A correlation was found between the AAC and the RS, between the two parameters and between the length and width of grains, likely resulting from correlation selection according to human preferences. Eleven associations on seven chromosomes were discovered for RS, while five SNPs on chromosome 6 were associated to AAC. Candidate genes and quantitative trait loci (QTLs) previously identified as affecting RS and AAC were co-located with six associations, validating the analysis. The present work represents a valuable source for future breeding programs aimed at improving rice grain quality by selecting the proper AAC according to market requests and by increasing RS amount in seeds.

These four outstanding papers are highly valuable contributions to *Plants*. On behalf of the *Plants* Editorial Board, we would like to congratulate these four teams for their excellent work. In recognition of their accomplishments, each team will receive a certificate and a cash award of 500 CHF plus a waiver to enable them to publish a paper free of charge in 2021.

We would like to take this opportunity to thank all the nominated research groups of the above exceptional papers for their contributions to *Plants*, and thank the *Plants* Editorial Board for voting and helping with this “Best Paper Award”.

The Editorial Board and Editorial Staff at *Plants* are committed to meeting the needs of our research community by providing constructive and timely reviews of all quality manuscripts submitted and providing an open access journal for the broad dissemination of your findings. Please consider submitting your work to *Plants*, and we look forward to considering your paper as a *Plants* Best Paper in the future.
